# The impact of different flexible substrates on the photothermal reduction quality of graphene oxide†

**DOI:** 10.1039/d4na00385c

**Published:** 2024-07-17

**Authors:** Matheus Guitti Bonando, Gabriel Monte Mór Moreira, Nathália Maria Moraes Fernandes, David Steinberg, Alisson Ronieri Cadore, Cecília de Carvalho Castro Silva, Lúcia Akemi Miyazato Saito

**Affiliations:** a Mackenzie School of Engineering, Mackenzie Presbyterian University Rua da Consolação, 896, CEP: 01302-907 São Paulo/SP Brazil lucia.saito@mackenzie.br; b Instituto de Ciências Ambientais, Químicas e Farmacêuticas, Universidade Federal de São Paulo, Laboratório de Química de Calixarenos, Espectroscopia Molecular e Catálise Brazil; c Brazilian Nanotechnology National Laboratory (LNNano), Brazilian Center for Research in Energy and Materials (CNPEM) Campinas/SP Brazil; d Mackenzie Institute for Research in Graphene and Nanotechnologies (MackGraphe), Mackenzie Presbyterian Institute São Paulo/SP Brazil

## Abstract

In this work, we demonstrate the impact of the photothermal reduction quality of graphene oxide (GO), which is affected by the material composition, roughness, and thermal properties of the membrane substrates. We show high efficiency reduced graphene oxide (rGO) conversion by applying a 405 nm pulsed laser in ambient conditions onto different flexible substrates. Three filter membranes, such as nylon, cellulose acetate, and nitrocellulose, are used as rGO thin film substrates, achieving sheet resistance of 51 ± 2, 58 ± 3, and 620 ± 40 Ω sq^−1^, respectively, which has been the lowest resistance reported in ambient conditions. Finally, we demonstrate that such flexible materials can be applied as temperature sensors ranging from 35 °C to 100 °C. The best sensitivity is achieved using nylon membranes, showing a smoother rGO surface and lower defect density.

## Abstract 2

This work shows the lowest reported sheet resistances obtained by laser-reduced graphene oxide in ambient conditions for nylon and cellulose acetate substrates (51 ± 2 and 58 ± 3 Ω sq^−1^) by optimizing the photothermal reduction of graphene oxide.

## Graphical abstract

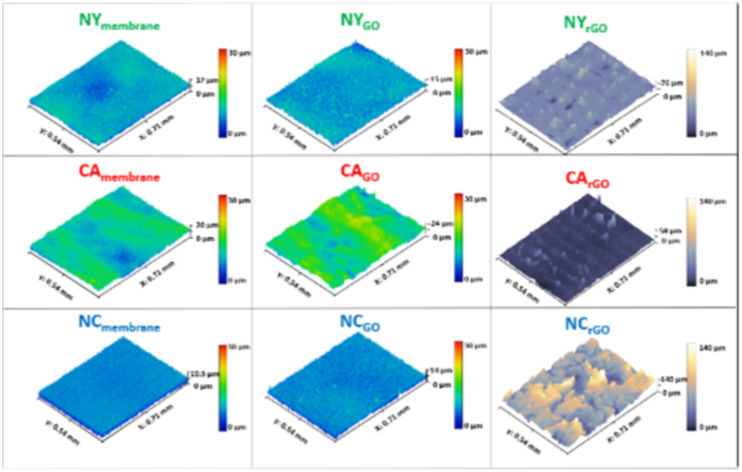

## Introduction

There is currently a significant innovation in the electronic devices field due to new emerging materials, such as two-dimensional (2D) materials, allowing several standardization techniques to develop flexible, wearable, and easy production of devices.^1,2^ Among these emerging materials, a class of materials that have gained attention is carbon materials, like carbon-based inks, which can optimize the electrical material properties and the resulting behavior of the final device.^3^ Graphene and its derivates are a great highlight that has been widely researched for various applications, including wearable devices,^4^ electrochemical sensors,^2^ supercapacitors,^5^ and temperature sensors,^6^ among many other applications.^5^ Graphene consists of a single layer of carbon atoms, it is seven times lighter than air, has high electrical conductivity (108 S m^−1^), thermal conductivity (5300 W m^−1^ K^−1^), high Young's modulus, high flexibility (1 TPa) and high surface area (2600 m^2^ g^−1^).^7^ However, the integration of graphene presents some challenges, like its handling, large-scale production, and transfer process, which uses several chemical reagents during its process.^8,9^

Aiming for low-cost and large-scale application of graphene in electronics, reduced graphene oxide (rGO) is an interesting strategy. Graphene oxide (GO) dispersions have characteristics that permit their high adhesion on different substrates due to their hydrophilic nature, allowing ease of handling and the achievement of thin films with high control of thickness. Although GO is an insulating material, it can suffer a reduction process, where oxygenated functional groups can be partially removed from their structure, thus restoring sp^2^ hybridization and its conductivity.^10^ Typically, the GO reduction process occurs by either thermal or chemical processes. One that has gained prominence is the photoreduction process^2,11^ due to the possibility of manufacturing electrically conductive patterns in applications that require dry material. Another benefit is that there is no need for solvents or purification steps, making it a good technique for green chemistry^12^.

The photoreduction process is divided into two processes: photothermal and photochemical.^5^ The photothermal process occurs when the wavelength is higher than 390 nm. The absorbed laser is converted into localized heat, and this high temperature causes weak breaks of the oxygenated functional groups in GO (–COOH and –OH). On the other hand, when the wavelength is lower than 390 nm, the photochemical process occurs due to the high energy of the photon. In this case, there is an immediate breakage of the chemical bonds of the oxygenated functional groups without significant heating of the substrate.^5,12^ The process of laser reduction of GO presents many variables that can affect the rGO quality. When analyzing the laser parameters, the spot size, the engraving speed, the frequency, average power, and the chosen wavelength can influence the results in the quality of the rGO. This aspect of photothermal reduction by varying the laser parameters was verified in previous work by our group.^13^

Another parameter that may influence the GO reduction process is the substrate used to perform this process. The substrate is usually chosen according to a specific application. Nevertheless, to the best of our knowledge, no work has investigated the influence of the substrates on the reduction of GO thin films. Nevertheless, it is plausible to expect that the changes in the membrane material composition, roughness, and thermal properties of the chosen material would impact the quality of the rGO film. Herein, we studied the electrical and thermal properties of rGO thin films obtained *via* laser reduction method from vacuum-filtrated GO films at different membranes substrates.

## Experimental

### Preparation of graphene oxide

The graphene oxide (GO) suspension was prepared using the modified Hummers process with three days of oxidation.^14,15^ Thus, the materials used were 0.50 g of graphite (Nacional do Grafite, Brazil – Graflake 9980 G, purity of 99,0% of carbon) and 0.38 g of NaNO_3_, to which 33.8 mL of concentrated H_2_SO_4_ were added under constant stirring and at ice bath. Within one hour, 2.25 g of KMnO_4_ was added. After cooling for 2 hours, the dispersion was stand for three days at room temperature and gently agitated until obtain a viscous liquid. Then, 1 L of a solution of H_2_SO_4_ (99.6 mL H_2_O to 0.28 mL H_2_SO_4_) was added for 1 hour. After this period, the liquid was continuously stirred for another 2 hours. Then, 1.5 mL of 30% (w/v) hydrogen peroxide was slowly added (Sigma-Aldrich). The prepared GO dispersion was washed three times with a 10% hydrochloric acid aqueous solution (Synth, 37% purity) to finish the process. The GO dispersion was purified using dialysis bags (porosity of 12 kDa) in deionized water until the residual GO water reached a pH of 5.0. Next, to remove silica residues from the GO dispersion, due to the type of graphite employed in the synthesis process, as previously observed in the study performed by R. Jalili *et al.*^16^ and M. A. Santos *et al.*,^17^ a solution of 0.1 M of NaOH was gradually added to it under magnetic stirring (1000 rpm) every 5 minutes until a pH equal to 11 was obtained.

### Vacuum filtration system and substrates

**Fig. 1:**
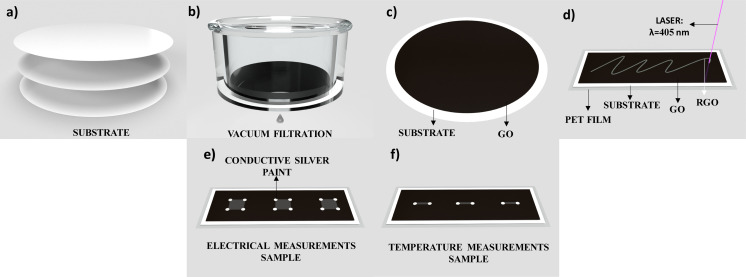
Schematic representation of the photoreduction process of GO films, and preparation of the rGO temperature sensors. (a) Substrates of Nylon (Ny), Cellulose Acetate (CA) and Nitrocellulose (NC) with 47 mm diameter and 0.22 μm pore size, (b) filtration of the GO dispersion through the selected membrane under vacuum, (c) thin GO film on the selected membrane surface, (d) reduction of GO film on the selected membrane by laser-scribing on PET film, (e) rGO patterns for electrical and (f) temperature sensors analysis.

Fig. 1 shows the schematic representation of the GO photoreduction process. The substrates of Fig. 1a are filtration membranes of Nylon (Ny), Cellulose Acetate (CA), and Nitrocellulose (NC) with diameter of 47 mm and pores of 0.22 μm. For the preparation of GO thin films on the filtration membrane surface, a volume of 3.5 mL with GO concentration of 5.6 mg mL^−1^ was filtered on each membrane in a vacuum filtration system (Fig. 1b) for 80 minutes, which the appearance of the film became visually dry and uniform. The samples were left to dry completely at room temperature for one day (Fig. 1c). Other variations in volume and concentration were performed to optimize the rGO thin film with low resistance and low defects.^13^ We kept the optimized GO volume and concentration during the tests performed for this paper. Three samples of each substrate from different batches were used to verify the reproducibility of the experiments, and the results remained stable even after more than one year since the first measurement.

### Photoreduction process

The photoreduction process was performed using a commercial INSMA laser engraving system with a 405 nm pulsed laser operating at a frequency of 282 Hz. These parameters were fixed throughout the procedure. The light scribe technology enabled the design of patterns through digital drawing (Fig. 1d) with a peak power of 2.56 W, an average power of 300 mW, a pulse width of 416 μs, a step of 16.5 mm s^−1^, and a spot size of 120 μm, which is the optimized setup 1 (OS1) for the reduction of GO on Ny and AC membranes. For the GO reduction on the NC membrane, the optimized setup 2 (OS2) has a peak power of 1.15 W, an average power of 78 mW, a pulse width of 240 μs and 17 mm s^−1^. The particular use of OS2 instead of OS1 was to avoid the flash point of the nitrocellulose membrane, thus keeping the same optimized parameters to obtain the lowest sheet resistance of the rGO. The membrane is placed on top of a PET film for heat dissipation, preventing wrinkling and damage during the reduction process.

### Electrical characterization

The electrical sheet resistance (*R*_s_) was characterized at 25 mm^2^ rGO patterns with silver electrical contact using micro-positioners from a Keysight B1500A Semicondutor Device Analyzer (Fig. 1e).

### Surface characterization

Confocal laser scanning microscopy (CLSM) was performed using a commercial Keyence VK-X200 system with a 20× objective (NA = 0.95). A 408 nm wavelength laser was used to characterize the roughness and the images, which allowed to visualize in detail the appearance of GO thin films on the surface of each membrane and after the photoreduction process on the rGO surface. The Raman spectroscopy measurements were performed using a 532 nm laser with 2 mW of power, 600 gr/nm grating, and a 10× objective lens of Witec UHTS 300 Raman spectrometer to evaluate the reduction efficiency. All single spectra were collected for 7 s and three accumulations. Also, we use a FLIR C3-X thermal camera to capture the temperature of the laser incident on the surface of the samples. The high-resolution spectra of the XPS measurements were performed with ten scans, spot size of 300 μm, pass energy of 50 eV, energy step size of 0.10 eV, and Dwell time of 50 ms of the K-alpha XPS (Thermo Scientific), using a monochromatic source with Al anode, Kα energy of 1486 eV.

### Temperature sensor characterization

The temperature sensor was characterized using a hotplate (TE-038/2-MP) by increasing the temperature with a step of 1 °C, stabilization time of 5 minutes, measuring the electrical resistance for each temperature, and a FLIR C3-X thermal camera was used to confirm the temperature of the surface device. The samples were prepared using 1 mm × 5 mm patterns on top of a thin polyethylene terephthalate (PET) film (Fig. 1f) to avoid any wrinkling during the reduction process and any damage to the substrates. Conductive silver paint was used as an electrical contact, and the measurements were made in three samples for each substrate, increasing the temperature from 35 °C to 100 °C.

## Results and discussions

**Fig. 2:**
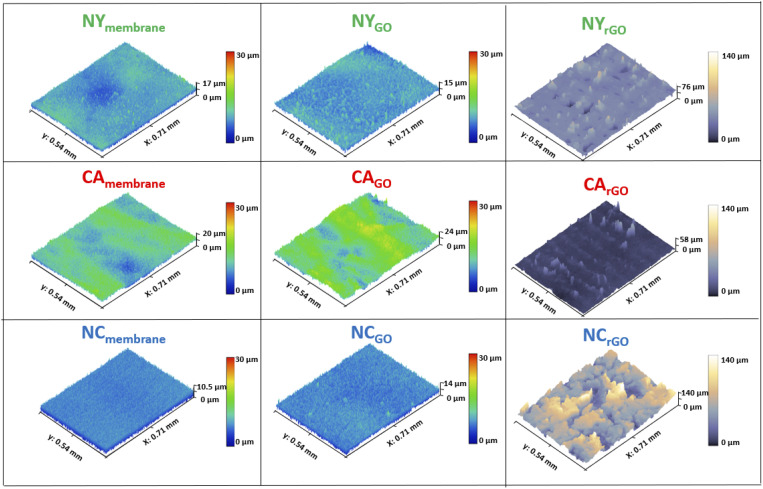
3D models of surface characterization of each sample by confocal laser scanning microscopy. Surface images of pure membrane (1st column), GO surface (2nd column), and rGO surface (3rd column) obtained with photoreduction process using OS1. Membranes: Nylon (Ny) in green, Cellulose Acetate (CA) in red, and Nitrocellulose (NC) in blue.

The morphology and topography of the surface samples were analyzed and characterized by CLSM measurements. Fig. 2 shows the CLSM images, which consist of surface membranes of Nylon (Ny) in green border images and trace, Cellulose Acetate (CA) in red, and Nitrocellulose (NC) in blue, respectively. The first figure column is the pure membranes before the vacuum filtration process. The second column presents the GO surface image after the filtration process. Finally, the third column shows the rGO surfaces after the photoreduction process, following the image sequences from left to right. By analyzing the images, we observed that the membrane surfaces without GO films showed a variation in their roughness. NC membrane was the least rough.

On the other hand, CA was the roughest one, and it directly influenced how GO flakes settle on the material's surface during the vacuum filtration process. Depending on the surface's roughness, gaps can form under the flakes. Table 1 shows those analyses' root mean square roughness.

**Table 1:** Root mean square roughness of the membranes Nylon (Ny), Cellulose Acetate (CA), and Nitrocellulose (NC)

	Roughness Rq (μm)		
	Membrane	GO	rGO
Ny	1.66 ± 0.34	1.88 ± 0.48	6.73 ± 0.81
CA	2.95 ± 0.52	2.78 ± 0.25	2.96 ± 0.42
NC	1.00 ± 0.04	1.05 ± 0.13	22.57 ± 0.91

This analysis of Table 1 shows that the membrane surfaces (1st column) have similar roughness than those with GO films (2nd column). After the GO filtration on the surface of the membranes, it was observed that the GO film tends to be uniform with its flakes settling through the material's roughness, thus showing a smoother variation on its surface. This roughness variation of the GO film can directly influence the reduction process since the oscillation on its surface during the laser movement along the sample will change the spot size area, thus altering the energy density in the photoreduction process, which impacts the degree of reduction. Another essential aspect being considered is the larger gaps under the flakes. During the reduction process, the rGO tends to detach from the substrate thus obtaining a non-uniform film with more significant defects and, in turn, impacting both the charge transport and the sheet resistance parameters.^18^

The flash point of the NC membrane is around 200 °C. Thus, in the reduction process with OS1, the temperature reaches values above 200 °C. Therefore, the GO film was burned along the NC membrane, causing an ablation of the GO during the reduction process. Each membrane dissipates the heat on rGO from the laser in different manners. Since it is a photothermal reduction, the laser absorbed by the GO is converted into localized heat to reduce the GO. Because of the influence of the substrate on the heat conductivity, the temperature achieved during the reduction process on the Ny membrane was 46.5% lower than the NC membrane and 29.7% lower than the CA membrane. Therefore, these results showed that each heat conductivity affected the reduction process in each membrane.

**Fig. 3:**
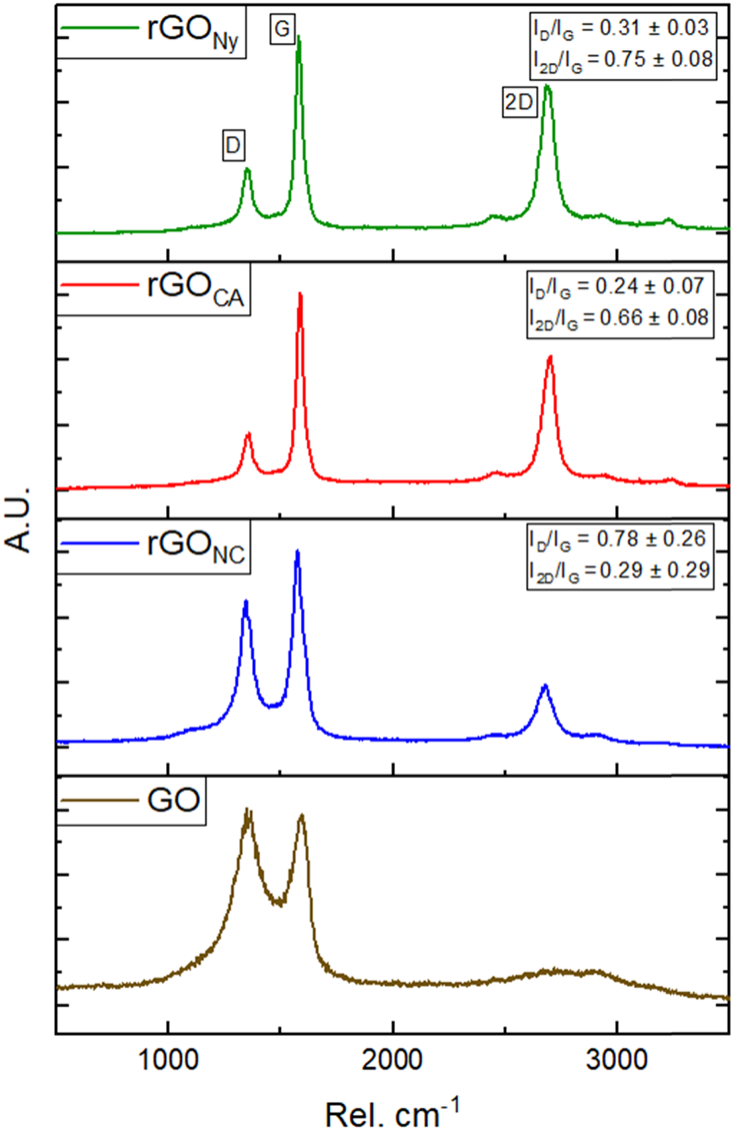
Raman spectra of rGO film in each membrane used as substrate compared to the GO Raman spectra.

To confirm the effectiveness of the laser's reduction degree of rGO, Raman spectra were taken at different parts of the graphene derivatives samples in the different membrane substrates, as shown in Fig. 3.

The Raman spectra of the GO samples, before and after the reduction process and independent of the substrate, indicated the presence of D-band and G-band peaks centered approximately at 1350 cm^−1^ and 1580 cm^−1^, respectively, and may have a slight variation in the position after reduction. The D-band is correlated with defects in the material's graphitic structure (sp^2^ bonds) and incomplete bonds at the edges. The G-band is associated with the stretching modes of the C=C bonds, which are contained in the material's structure, and its enlargement indicates more significant heterogeneity or structural disorders.^19^

The 2D band's peak, centered at approximately 2700 cm^−1^, is correlated with the second-order scattering of the D-band. It also indicates the number of graphene layers, which is crucial for proving the efficiency of the reduction process.^20^

The ratio intensity between D and G bands (*I*_D_/*I*_G_), is a qualitative tool to evaluate the structural defects in the material.^21^ If this ratio is greater than 1, then the sp^2^ hybridization has been interrupted, as many defects exist. Otherwise, when *I*_D_/*I*_G_ is less than 1, the material has fewer structural defects and a better graphitic network.^21^ The proportions of the intensities of the D and G peaks (*I*_D_/*I*_G_) and the 2D and G peaks (*I*_2D_/*I*_G_) in the Raman spectra of the surfaces reflect the defect density and the extent of surface graphitization before and after laser treatment. Ideally, the reduction process aims to achieve minimal defects and maximum effectiveness of the photoreduction and graphitization (low *I*_D_/*I*_G_ and high *I*_2D_/*I*_G_).^18^ In Fig. 3a, we can see the difference in the intensity of the G-band and 2D-band in each sample and its *I*_D_/*I*_G_ and *I*_2D_/*I*_G_ band ratios.

The higher *I*_D_/*I*_G_ = 0.78 ± 0.26 and lower *I*_2D_/*I*_G_ = 0.29 ± 0.29 ratios for rGO in the NC membrane were caused by the formation of porous reduced graphene oxide on its surface. Even though the CA samples had a higher surface roughness, the *I*_D_/*I*_G_ = 0.24 ± 0.07 and *I*_2D_/*I*_G_ = 0.66 ± 0.08 in this sample were similar to the Ny sample. This last one presented the highest *I*_D_/*I*_G_ = 0.31 ± 0.03 and *I*_2D_/*I*_G_ = 0.75 ± 0.08.

By measuring the full width at half maximum (FWHM) of the Lorentzian 2D band profile, we obtained 80 ± 1 cm^−1^, 82 ± 1 cm^−1^, and 99 ± 2 cm^−1^ for Ny, CA, and NC membranes, respectively, which are characteristic values for few-layer (Ny and CA) or multiple graphene layers (NC).

The restructuring of the graphitic plane can also be estimated by calculating the size of the crystalline domain (*L*_a_) in the structure by the following formula:^22^1
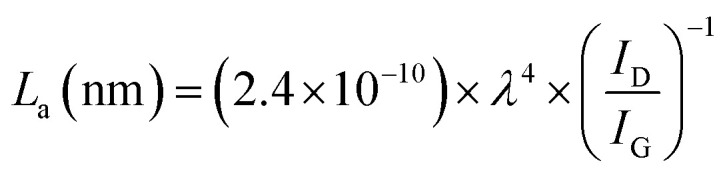
where *λ* is the wavelength of the laser (532 nm). This calculation was only obtained for rGO on the Ny (62.23 ± 4.96 nm) and AC membranes (88.29 ± 22.01 nm); except for NC, which the values were above 0.5.

**Fig. 4:**
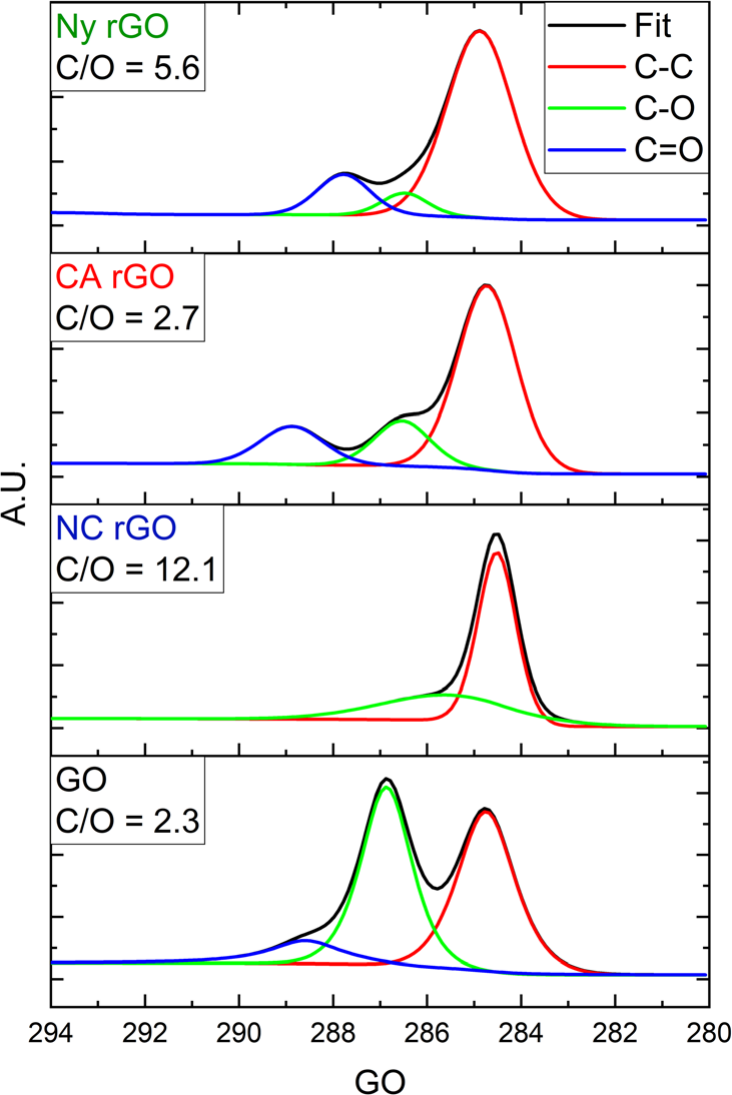
High-resolution C 1s XPS spectra of the rGO on Ny, CA, and NC membranes and GO film, respectively. In the inset, the C/O ratio for each sample is shown.

XPS measurements of the samples were made to improve the analysis of the degree of reduction. Fig. 4 shows the effectiveness of C–O and C=O groups reduction in rGO of all membranes.

As result, C/O ratios of 5.6, 2.7 and 12.1 were obtained for Ny, CA, and NC membranes, respectively. Although the NC membrane showed the best C/O ratio, indicating a better reduction, the formation of the porous reduced graphene oxide structure on its surface with high defects (as previously seen in the Raman analysis) also induced a higher electrical resistance than the other samples.

In the final step, we performed the characterization of rGO sheet resistance, given the previous optimized parameters. The objective was to achieve the lowest *R*_s_ in the experiments since the reduction process aimed to remove the oxygenated functional groups from the structure, restoring the sp^2^ hybridization of graphene in the material, thus increasing its electrical conductivity. Therefore, both concentration and volume of filtered GO for the formation of thin films in each membrane, as well as the laser's power, speed, and spot size, were optimized and adjusted until the sheet resistance was minimal, resulting in the OS1 and OS2 parameters. To verify the reproducibility of the experiment, measurements were performed on a total of nine samples with 25 mm^2^ area per membrane.

As result, the minimum sheet resistances (*R*_s_) of rGO on optimized laser conditions were 51 ± 2, 58 ± 3, and 620 ± 40 Ω sq^−1^ for Ny, CA, and NC membranes, respectively. For Ny and CA membranes, high-quality rGO was obtained with the photoreduction process using OS1. Those *R*_s_ are the lowest resistance reported for graphene oxide laser reduction in ambient conditions; for NC membrane, it was used OS2. The sheet resistance values were consistent with the Raman spectra results, showing the best (low *I*_D_/*I*_G_, high *I*_2D_/*I*_G_) and worst (high *I*_D_/*I*_G_, low *I*_2D_/*I*_G_) for Ny and NC samples, respectively.

Next, we show the influence of each substrate for ultrathin temperature sensor application. We studied the performance of each membrane as a function of the temperature; the normalized resistance change was introduced to assess the device's sensitivity. It is defined as Δ*R*/*R* where Δ*R* = *R* −*R*_0_, *R* is the real-time resistance, and *R*_0_ is the initial resistance. These measurements were performed with three samples of each membrane. The results were reproducible more than one year after the first measurement.

**Fig. 5:**
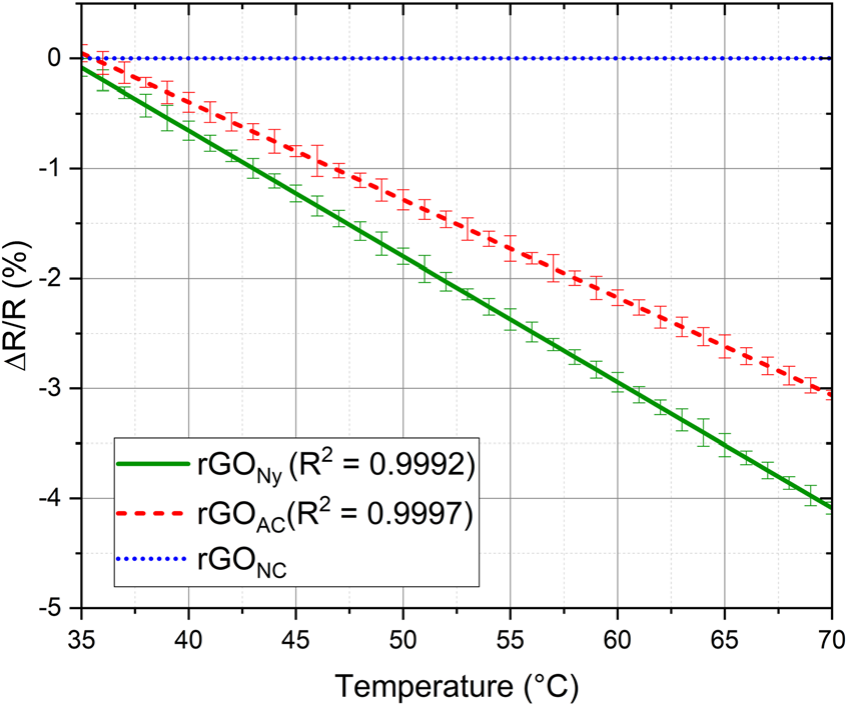
Response of temperature rGO sensors in different substrates.

The sensitivity performance of each temperature sensor is shown in Fig. 5. The rGO temperature sensitivity in each membrane can be explained by the thermal excitation of the carriers in the rGO. As the temperature increases, the carriers' probability to overcome the potential barrier rises, and the tunneling effect of the carriers between adjacent rGO layers increases. Therefore, the mobility of the rGO carrier boosts significantly as the temperature grows, which leads to a decrease in resistance,^23^ which means that the electrical modulation is not caused by the value of the initial sheet resistance itself, but by the thermal excitation. There is no resistance modulation for the sensor using rGO on the NC membrane despite the increase in the temperature; since the rGO on the NC membrane is porous, the thermal excitation does not occur in this sample for the high space gaps between the porous reduced graphene oxide flakes and the heat transmission along the surface can be highly decreased by the presence of air between the flakes, so the morphology of the material may cause the absence of modulation after the reduction. Otherwise, the Ny sample shows the best results compared to the CA sample, showing a higher sensibility. By returning to the initial temperature of 35 °C, the sheet resistance returns to the initial values with a slight increase of approximately 1–2%.

## Conclusions

In conclusion, our work showed the strong influence of different flexible substrates on the GO photothermal reduction process by using a laser of 405 nm. The nitrocellulose membrane reached its flash point, damaging the GO thin film, while the Nylon and Cellulose Acetate membranes had their best rGO parameters. In addition, we need to use different laser parameters to activate photoreduction on the nitrocellulose substrate. However, the minimum resistance is ten times higher with a porous reduced graphene oxide surface. By changing the substrate material, we could obtain the lowest reported sheet resistance for GO reduced by laser in ambient conditions, which also directly influenced the results of possible applications, as demonstrated in the temperature sensor.

## Data availability

The authors confirm that the data supporting this study's findings are available within the article and its supplementary material. Raw data supporting this study's findings are available from the corresponding author upon reasonable request.

## Author contributions

L. A. M. Saito and C. C. C. Silva conceived the idea and designed the research. M. G. Bonando, G. M. M. Moreira, and N. M. M. Fernandes performed the experiments. A. R. Cadore and D. Steinberg performed the AFM and CLSM measurements. M. G. Bonando and C. C. C. Silva performed MEV measurements. All the authors contributed to the data treatment and interpretation of the results. M. G. Bonando wrote the manuscript. All the authors reviewed the manuscript.

## Conflicts of interest

There are no conflicts to declare.

## Supplementary Material

NA-006-D4NA00385C-s001
