# Supplementary material for: The impact of different flexible substrates on the photothermal reduction quality of graphene oxide
Source: Nanoscale Adv. 2024 Jul 17;6(18):4604–10. doi: 10.1039/d4na00385c (PMC11385539; doi:10.1039/d4na00385c)
Supplement: NA-006-D4NA00385C-s001 [file NA-006-D4NA00385C-s001.pdf]

Electronic Supplementary Material (ESI) for Nanoscale Advances.
This journal is © The Royal Society of Chemistry 2024

Supplementary Information

The impact of different flexible substrates on the photothermal reduction quality of graphene oxide

Matheus Guitti Bonando,[a] Gabriel Monte Mór Moreira,[a] Nathalia Maria Moraes Fernandes,[a,b] David Steinberg,[a] Alisson Ronieri Cadore,[c] Cecília de Carvalho Castro Silva,[a,d] Lúcia Akemi Miyazato Saito,*[a,d]

[a] *Mackenzie School of Engineering, Mackenzie Presbyterian University, Rua da Consolação, 896, CEP: 01302-907, São Paulo/SP, Brazil*
[b] *Instituto de Ciências Ambientais, Químicas e Farmacêuticas, Universidade Federal de São Paulo, Laboratório de Química de Calixarenos, Espectroscopia Molecular e Catálise*
[c] *Brazilian Nanotechnology National Laboratory (LNNano), Brazilian Center for Research in Energy and Materials (CNPEM), Campinas/SP, Brazil.*
[d] *Mackenzie Institute for Research in Graphene and Nanotechnologies (MackGraphe), Mackenzie Presbyterian Institute, São Paulo/SP, Brazil.*
**e-mail: lucia.saito@mackenzie.br*

Substrate	Rougness Rq (µm)			Roughness Rq Mean (µm)
Ny	1.18	1.90	1.90	1.66 ± 0.34
CA	3.47	2.44	-	2.95 ± 0.52
NC	1.06	0.99	0.97	1.00 ± 0.04
Ny-GO	2.01	1.24	2.38	1.88 ± 0.48
CA-GO	2.53	3.13	2.70	2.78 ± 0.25
NC-GO	1.19	1.09	0.87	1.05 ± 0.13
Ny-rGO	8.10	6.38	5.70	6.73 ± 0.81
CA-rGO	3.09	3.40	2.39	2.96 ± 0.42
NC-rGO	23.86	21.86	22.00	22.57 ± 0.91

Table 1 - Data of root mean square roughness for the membranes Nylon (Ny), Cellulose Acetate (CA), and Nitrocellulose (NC) for different samples.

Substrate	Rs (Ω/sq)			Rs Mean (Ω/sq)
Ny-rGO	49	50	54	51 ± 2
CA-rGO	55	57	62	58 ± 3
NC-rGO	572	632	664	620 ± 40

Table 2 – Data of electrical sheet resistance for the membranes Nylon (Ny), Cellulose Acetate (CA), and Nitrocellulose (NC) for different samples.

	rGO-Ny		
	ΔR/R (%)		
**Temperature**	**Sample 1**	**Sample 2**	**Sample 3**
35	0	0	0
36	-0.24092	-0.22092	-0.06092
37	-0.31225	-0.36225	-0.14225
38	-0.47397	-0.44397	-0.35397
39	-0.63763	-0.68763	-0.44763
40	-0.69156	-0.79156	-0.51156
41	-0.86433	-0.87433	-0.68433
42	-1.02018	-0.94018	-0.84018
43	-1.0736	-1.0536	-0.9536
44	-1.16863	-1.27863	-1.05863
45	-1.30392	-1.33392	-1.18392
46	-1.46947	-1.48947	-1.31947
47	-1.49631	-1.57631	-1.35631
48	-1.5633	-1.5933	-1.4233
49	-1.68951	-1.70951	-1.51951
50	-1.886	-1.876	-1.656
51	-2.00353	-1.89353	-1.82353
52	-2.05122	-2.10122	-1.82122
53	-2.20837	-2.15837	-2.00837
54	-2.28579	-2.24579	-2.16579
55	-2.43348	-2.38348	-2.25348
56	-2.51145	-2.57145	-2.34145
57	-2.57968	-2.66968	-2.44968
58	-2.73819	-2.76819	-2.63819
59	-2.81697	-2.79697	-2.66697
60	-2.92603	-2.96603	-2.78603
61	-3.12537	-3.09537	-2.92537
62	-3.16498	-3.27498	-3.09498
63	-3.39487	-3.34487	-3.23487
64	-3.40504	-3.47504	-3.23504
65	-3.63548	-3.56548	-3.34548
66	-3.71621	-3.71621	-3.49621
67	-3.85723	-3.83723	-3.71723
68	-3.91852	-3.93852	-3.76852
69	-4.0101	-4.0701	-3.9301
70	-4.12196	-4.18196	-3.96196

Table 3 – Data of the temperature by the electrical resistance for the rGO on Ny membrane.

	rGO-CA		
	ΔR/R (%)		
**Temperature**	**Sample 1**	**Sample 2**	**Sample 3**
35	0	0	0
36	-0.03602	-0.13602	0.05398
37	-0.08219	-0.29219	-0.06219
38	-0.12851	-0.36851	-0.22851
39	-0.30498	-0.37498	-0.26498
40	-0.40159	-0.57159	-0.33159
41	-0.37836	-0.59836	-0.46836
42	-0.53527	-0.70527	-0.48527
43	-0.63234	-0.76234	-0.56234
44	-0.67956	-0.83956	-0.68956
45	-0.74693	-0.99693	-0.79693
46	-0.87445	-0.99445	-0.91445
47	-0.84826	-1.14826	-0.84826
48	-1.10994	-1.20994	-1.04994
49	-1.10792	-1.26792	-1.16792
50	-1.25605	-1.42605	-1.18605
51	-1.27434	-1.46434	-1.26434
52	-1.35277	-1.55277	-1.37277
53	-1.53137	-1.66137	-1.48137
54	-1.63011	-1.77011	-1.52011
55	-1.60902	-1.77902	-1.68902
56	-1.73807	-1.94807	-1.67807
57	-1.86729	-1.98729	-1.88729
58	-1.94666	-2.17666	-1.89666
59	-1.98619	-2.14619	-2.08619
60	-2.11587	-2.34587	-2.12587
61	-2.13571	-2.30571	-2.25571
62	-2.35571	-2.44571	-2.27571
63	-2.35587	-2.57587	-2.37587
64	-2.53619	-2.68619	-2.47619
65	-2.55667	-2.73667	-2.56667
66	-2.60731	-2.85731	-2.67731
67	-2.7881	-2.8881	-2.6981
68	-2.88906	-3.04906	-2.86906
69	-2.91018	-3.10018	-2.93018
70	-3.03146	-3.16146	-3.00146

Table 4 – Data of the temperature by the electrical resistance for the rGO on CA membrane.

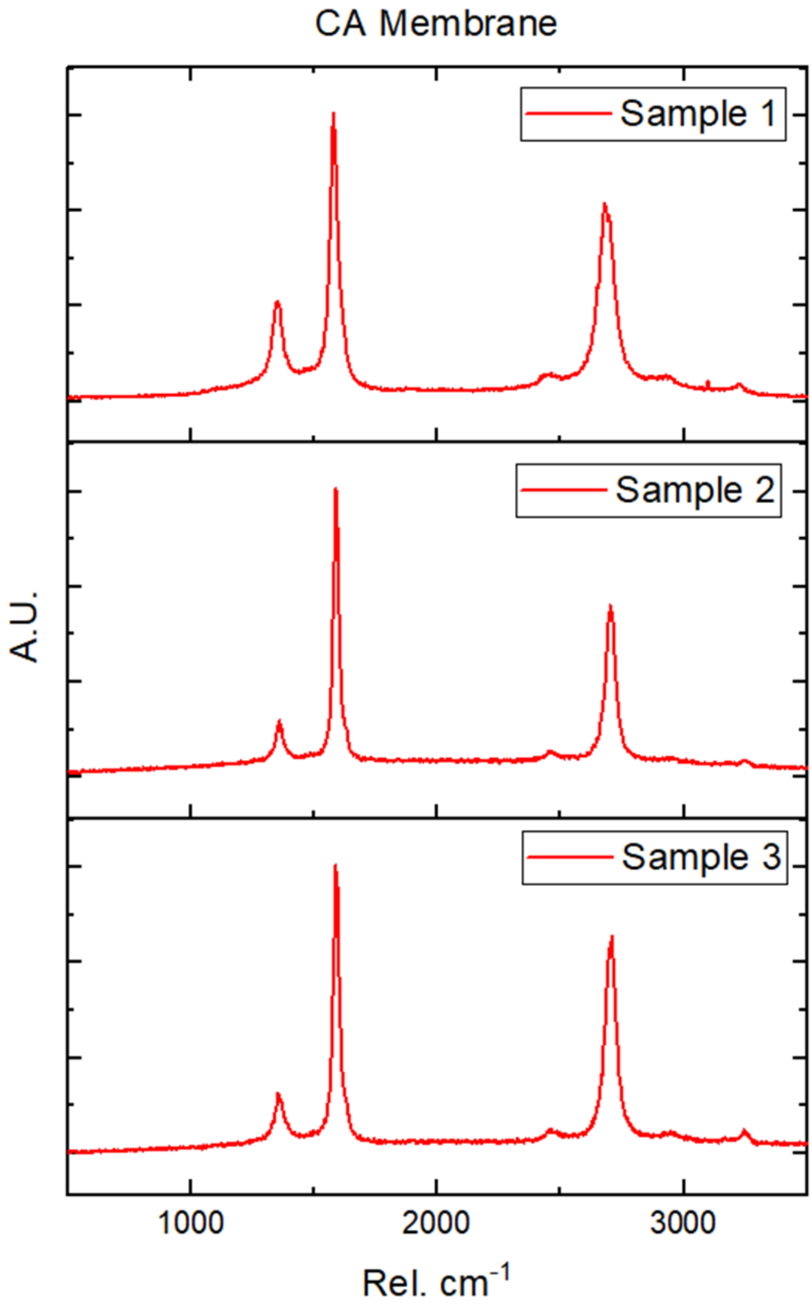

Figure 1 - Data of Raman Spectra of rGO for different samples on CA membrane.

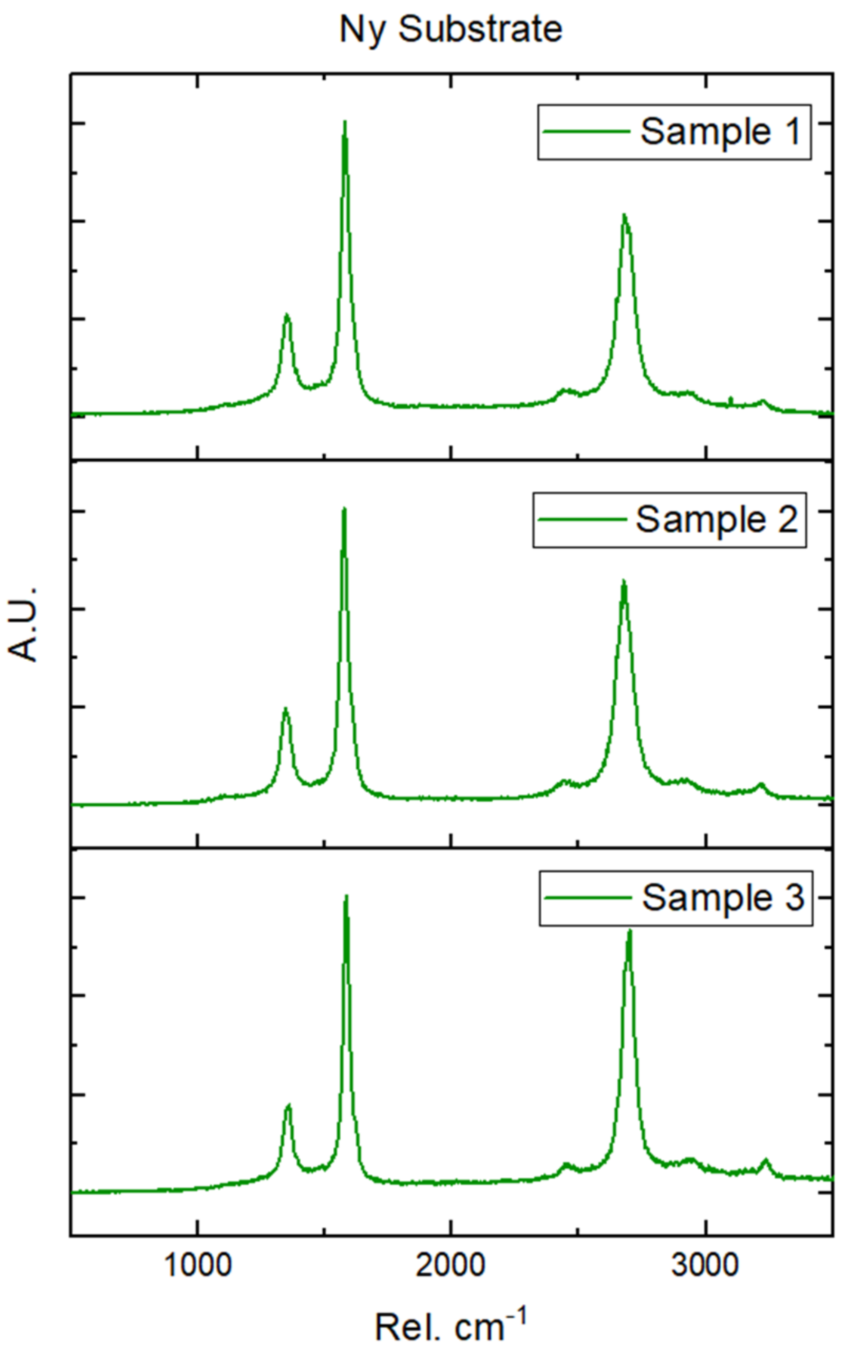

Figure 2 - Data of Raman Spectra of rGO for different samples on Ny membrane.

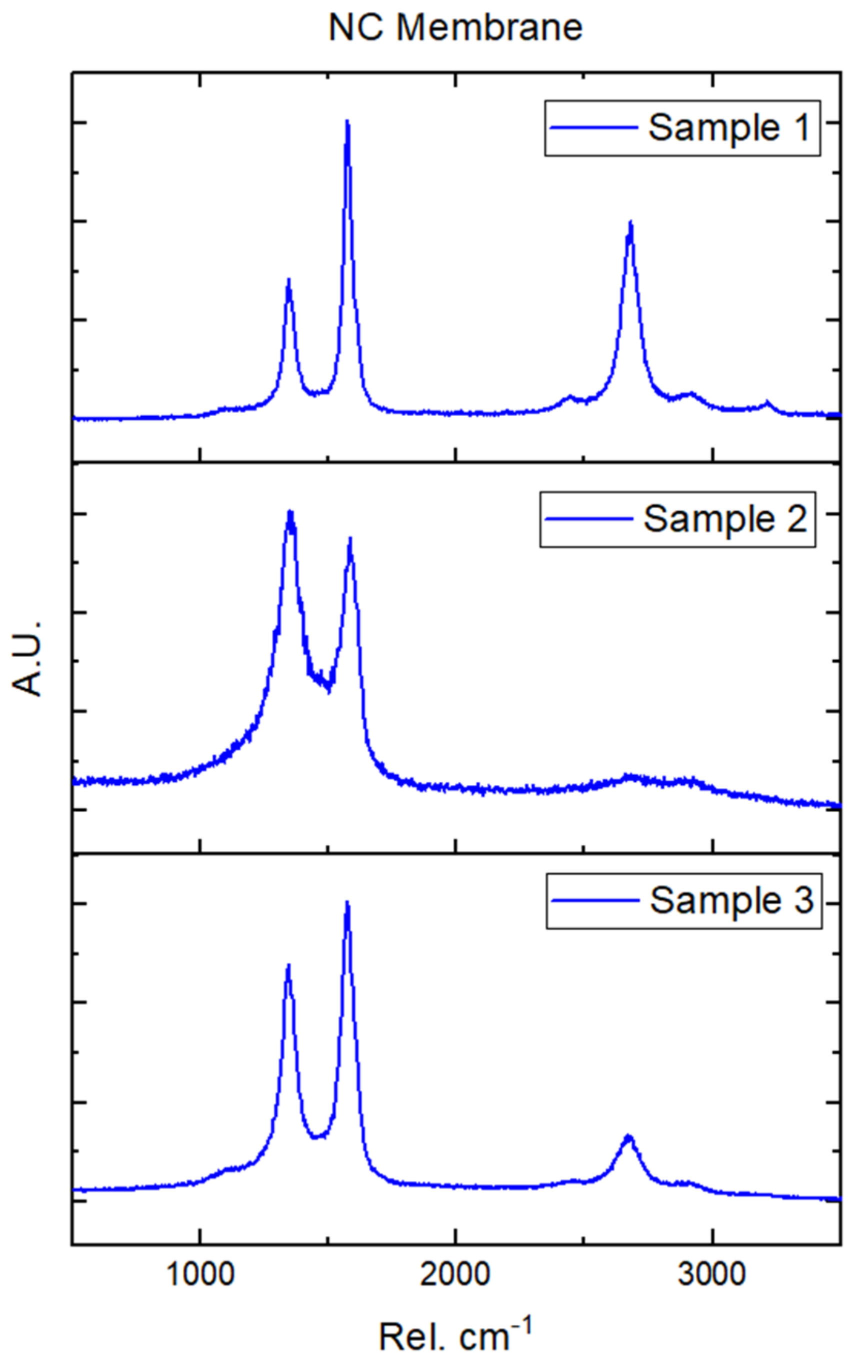

Figure 3 - Data of Raman Spectra rGO for different samples on Nitrocellulose (NC) membrane.
